# Online-Initiated Contact and Child Sexual Abuse Severity: A Forensic Cross-Sectional Study

**DOI:** 10.3390/children13081097

**Published:** 2026-08-18

**Authors:** Fatmagül Aslan, Halit Canberk Aydogan, Hacer Yaşar Teke, Işıl Pakiş, Deniz Özel

**Affiliations:** 1 Clinic of Forensic Medicine, Antalya Training and Research Hospital, Health Sciences University, Antalya 07100, Türkiye; drfatmagulaslan@yahoo.com; 2 Department of Forensic Medicine, Ordu University Training and Research Hospital, Ordu 52200, Türkiye; hacer.hgulderen2004@gmail.com; 3 Department of Forensic Medicine, School of Medicine, Acıbadem Mehmet Ali Aydınlar University, İstanbul 34752, Türkiye; isilpakis@yahoo.com; 4 Statistical Consulting Application and Research Center, Akdeniz University, Antalya 07070, Türkiye; denizozel@akdeniz.edu.tr

**Keywords:** child sexual abuse, online-initiated contact, online grooming, technology-facilitated abuse, cyber harassment, adolescents, forensic medicine

## Abstract

**Highlights:**

**What are the main findings?**

- Among 1511 forensic child sexual abuse cases, first contact between the victim and the perpetrator occurred online in 6.0% of cases, and these victims were predominantly adolescent girls.
- Penetrative abuse was approximately twice as prevalent when first contact occurred online (54.9% vs. 26.9%; *RR* = 2.04), and the perpetrator was most often perceived by the child as a friend or boyfriend.

**What are the implications of the main findings?**

- Perceived romantic relationships in online-initiated cases are consistent with grooming dynamics and remain legally and developmentally non-consensual.
- Systematic documentation of online contact histories may strengthen forensic evaluation and child protection responses.

**Abstract:**

**Background/Objectives:** Digital technologies have created new pathways through which perpetrators can initiate contact with children, yet evidence directly comparing child sexual abuse (CSA) cases according to the mode of first contact between the victim and the perpetrator remains limited. This study compared CSA cases in which the victim first met the perpetrator online with cases in which first contact occurred face-to-face and examined whether online-initiated contact was associated with a higher prevalence of penetrative abuse. **Methods:** We retrospectively reviewed 1511 CSA cases evaluated at a tertiary-care Child Monitoring Center in Turkey (2017–2018). Cases were classified by mode of first contact as online-initiated (*n* = 91, 6.0%) or face-to-face (*n* = 1420, 94.0%); the face-to-face group served as a comparison group defined by mode of contact rather than a non-exposed control group. Groups were compared using chi-square tests with Bonferroni-adjusted post hoc analyses; effect sizes were expressed as Cramér’s *V* and risk ratios (*RRs*). **Results:** Victims in the online-initiated group were predominantly female (89.0% vs. 79.4%; *p* = 0.027) and aged 12–18 years (79.2%). The perpetrator was most often described by the child as a friend or boyfriend (81.3% vs. 34.2%; *p* < 0.001), a pattern consistent with grooming dynamics. Penetrative abuse occurred in 54.9% of online-initiated versus 26.9% of face-to-face cases, an approximately twofold difference (*RR* = 2.04; *p* < 0.001). **Conclusions:** In this forensic sample, online-initiated contact was associated with a distinct case profile and more frequent penetrative abuse. Because the data derive from cases referred by judicial authorities for forensic evaluation rather than from adjudicated cases, the findings are associative and cannot be generalized to all CSA; nevertheless, they support the systematic documentation of online contact histories in forensic evaluation and child protection practice.

## 1. Introduction

Child sexual abuse (CSA) is any act performed by an adult or a significantly older child for sexual gratification involving a child below the legal age of consent [1], and sexual violence against children constitutes a substantial global burden [2]. Throughout this article, the term “child” is used in its legal sense to denote any person under 18 years of age, in line with the United Nations Convention on the Rights of the Child; the terms “adolescent” and “minor” are used where developmental distinctions within this legal category are relevant. Over the past two decades, the widespread adoption of internet-connected devices has transformed the contexts in which such abuse can occur: digital technologies now play a role both in the initiation of abusive contact and in the perpetration and extension of the abuse itself [3,4]. Nationally representative data from the United States indicate that a considerable proportion of youth experience online or technology-facilitated sexual offenses during childhood [5].

Several related but distinct constructs describe harmful online interactions directed at children. Cyber harassment refers to the use of internet-based tools such as e-mail, instant messaging, and social media to disturb, threaten, or stalk a person [6]. Cybergrooming, by contrast, denotes a psychologically manipulative process in which trust is built with a child in order to facilitate sexual abuse; it may culminate in online and/or offline victimization but should not be equated with the abuse itself [7]. Recent systematic reviews have characterized the victimization side of cybergrooming, including prevalence rates, risk factors, and outcomes [7], as well as the perpetrator side, including offender characteristics, strategies, and typologies [8]. In an early conceptual account, Durkin identified four principal ways in which individuals with a sexual interest in children may misuse the internet: trafficking child sexual abuse material, locating children to abuse, engaging in inappropriate sexual communication with children, and networking with other offenders [9].

A growing body of research indicates that online and offline sexual victimization are intertwined rather than separate phenomena. In their influential analysis of internet-initiated sex crimes, Wolak and colleagues showed that such offenses rarely conform to the stereotype of a violent or deceptive stranger; instead, they most often resemble statutory offenses in which adult offenders openly develop relationships with adolescents who are aware that they are communicating with adults [10]. Retrospective survey data similarly show that a substantial proportion of young people who conversed with adult strangers online as minors experienced sexual solicitation or grooming [11]. Moreover, harmful online interactions are frequently perpetrated by individuals known to the young person offline rather than by online-only contacts [12], and technology is increasingly embedded in abuse that occurs within pre-existing relationships [4].

The theoretical framework of the present study is Routine Activity Theory (RAT). As originally formulated by Cohen and Felson, RAT proposes that the likelihood of criminal victimization increases when a motivated offender and a suitable target converge in time and space in the absence of capable guardianship [13]. The framework has since been extended to digital environments, in which online platforms can broaden offenders’ access to potential victims while weakening conventional forms of supervision [14,15]. Consistent with this perspective, risky internet use and the online disclosure of personal information have been associated with elevated victimization risk among adolescents [16].

Despite this progress, most empirical work has concentrated on online interactions themselves, including the strategies offenders use in digital environments [17,18,19] and the prevalence and characteristics of online sexual harassment and solicitation [20,21]. Comparatively few studies have traced the transition from virtual first contact to hands-on sexual abuse [22], and forensic case series that directly compare abuse characteristics according to how first contact occurred remain scarce.

The present study addresses this gap using a large forensic sample of CSA cases referred by judicial authorities for evaluation at a Child Monitoring Center (CMC) in Turkey. We conceptualized the mode of first contact (online versus face-to-face) as a contextual characteristic of the abuse pathway. Accordingly, this study aimed (i) to compare the sociodemographic and contextual characteristics of online-initiated and face-to-face-initiated CSA cases, and (ii) to test whether the likelihood of penetrative sexual abuse was greater when first contact occurred online. We hypothesized that adolescents, particularly girls, would be overrepresented among online-initiated cases and that penetrative abuse would be more frequent in this group.

## 2. Materials and Methods

### 2.1. Setting: Child Monitoring Centers in Turkey

Child Monitoring Centers (CMCs) in Turkey provide a 24-h, multidisciplinary, on-call service for the evaluation and management of child abuse and neglect cases. These centers operate within a standardized national framework and are staffed by specially trained physicians and allied healthcare professionals, including social service specialists, psychologists, child development specialists, and nurses. For confidentiality purposes, the identity information of all cases admitted to CMCs is coded at the time of entry, and this coding system is used consistently across all forensic and clinical records. During data extraction, only coded study variables were transferred to the analytical dataset. Direct identifiers, including names, national identification numbers, addresses, and institutional file numbers, were not included in the dataset used for analysis.

### 2.2. Study Design and Participants

This retrospective cross-sectional study included all cases that underwent forensic medical examination and were officially registered at a tertiary-care CMC affiliated with the University of Health Sciences between January 2017 and December 2018 (*n* = 611 in 2017 and *n* = 900 in 2018; total *N* = 1511). All cases had been referred by judicial authorities, principally public prosecutor’s offices and law enforcement agencies, for forensic medical evaluation of suspected CSA; the designation “judicially reported” used throughout this article therefore denotes referral for such evaluation and does not imply that the allegations had been adjudicated or legally substantiated; final judicial outcomes were not available in the dataset. To improve clarity, the characteristics of the entire sample are described first in the Results, followed by comparisons between the contact groups. Because the dataset reflects cases recorded during 2017–2018, the findings may not fully capture more recent changes in digital platforms, online communication practices, or reporting behaviors.

### 2.3. Inclusion Criteria and Group Definitions

Cases were included if a formal request for a forensic medical examination evaluating findings related to physical and/or sexual abuse had been issued; in some cases, an additional psychiatric examination was requested by the public prosecutor. Eligible cases were then classified by the mode of first contact recorded in the case files:

1. Online-initiated contact group: the victim and the perpetrator first met through online platforms, whether their interaction subsequently remained online or progressed to in-person contact (*n* = 91).
2. Face-to-face contact group: first contact took place offline, whether or not the interaction later included online communication (*n* = 1420).

It should be emphasized that the face-to-face group constitutes a comparison group defined by the mode of first contact rather than a non-exposed control group; both groups consist of children evaluated for suspected sexual abuse. No cases were excluded from the study, as all eligible records contained sufficient information to classify the mode of first contact.

### 2.4. Data Collection and Variables

Case files systematically documented the manner in which the victim and the perpetrator first met. In addition to sociodemographic variables (age and gender), the following contextual variables were retrospectively extracted: the setting of the initial encounter, the nature and closeness of the victim–perpetrator relationship, the number of perpetrators involved, the location of the incident, substance use, presence of disability in the victim, pregnancy status, exposure to sex trafficking, the reporting source, and the type of abuse (penetrative versus non-penetrative).

Two operational clarifications are warranted. First, the perpetrator relationship categories (e.g., “boyfriend/friend”) reflect the relationship as described by the child and recorded in the case file. Because all victims were below the legal age of consent, any sexual contact occurring within such perceived relationships cannot be consensual in either legal or developmental terms; the label therefore describes the child’s perception of the relationship, which in online-initiated cases is likely to reflect grooming dynamics, and does not imply consensual contact. Second, the substance use variable describes the victim, not the perpetrator. In the CMC records, this field is a binary administrative entry coded positive when any use of a psychoactive substance, including tobacco and alcohol, appears at any point in the file, irrespective of frequency, severity, temporal relation to the incident, or toxicological confirmation, and no standardized screening instrument underlies the entry. Variables describing CMC procedures (e.g., forensic interview, family interview, temporary accommodation) were extracted to characterize the operational features of the center and were used descriptively only.

### 2.5. Statistical Analysis

Statistical analyses were performed using SPSS software, version 23.0 (IBM Corp., Armonk, NY, USA). The normality of distributions was assessed using the Kolmogorov–Smirnov test. Descriptive statistics were used to summarize the characteristics of the cases and the operational features of the CMC. Comparisons between the online-initiated and face-to-face contact groups were conducted using the Pearson chi-square test. In multi-cell contingency tables in which the chi-square test yielded statistically significant results, Bonferroni-adjusted post hoc analyses were applied to allow pairwise comparisons of column proportions. Effect sizes are reported as Cramér’s *V*, and the association between the mode of first contact and penetrative abuse is additionally expressed as a risk ratio (*RR*). Multivariable adjustment was considered but not performed for three reasons: the perceived victim–perpetrator relationship is plausibly an intermediate step on the grooming pathway linking online-initiated contact to abuse severity, and conditioning on a potential mediator carries a risk of overadjustment bias; the small size of the online-initiated group produced sparse cells across strata of age and perpetrator identity; and several candidate confounders were not available in the records. The risk ratio is accordingly presented as an unadjusted, descriptive measure of association. Statistical significance was defined as a two-tailed *p* value of less than 0.05.

### 2.6. Ethical Considerations

This retrospective study was conducted in accordance with the principles of the Declaration of Helsinki and all applicable regulations concerning the protection of personal data. Prior to study initiation, institutional permission was obtained from the national health authority, and the study was carried out at a tertiary-care hospital within the national health system in Turkey. Ethical approval was granted by the Ethics Committee of Health Sciences University Antalya Training and Research Hospital (Decision No. 2019-20/06). Access to the study data was restricted to the investigators responsible for the statistical analyses, and no third parties were granted access. All data were stored in encrypted, password-protected files to ensure confidentiality and data security. Given the retrospective design of the study and the use of fully anonymized forensic records, the ethics committee waived the requirement for obtaining additional parental consent and child assent.

## 3. Results

### 3.1. Characteristics of the Overall Sample

Of the *N* = 1511 cases examined, 1209 (80.0%) victims were female and 302 (20.0%) were male, corresponding to a male-to-female ratio of 1:4. The mean age of the victims was 12.80 ± 3.34 years, and the mean age of the perpetrators was 27.87 ± 15.25 years. The person reporting the event was most often a parent (*n* = 503, 33.3%), followed by teachers (*n* = 490, 32.4%), the children themselves (*n* = 168, 11.1%), and others, including healthcare personnel, police, relatives, and neighbors (*n* = 350, 23.2%). Penetrative abuse was documented in 432 cases (28.6%), pregnancy in 137 (9.1%), and exposure to sex trafficking in 13 (0.9%). The substance use variable, as documented in the victims’ case files, was coded positive in 1426 cases (94.4%); the operational definition of this variable is provided in Section 2.4. The characteristics of the overall sample are presented in Table 1.

During the evaluation process, temporary accommodation was provided at the CMC in 2.0% of cases. A forensic interview was conducted in 90.9% of cases, whereas a family interview was conducted in only 2.4% of cases. The procedures applied at the CMC, presented for descriptive purposes, are summarized in Table 2.

### 3.2. Comparison by Mode of First Contact

First contact occurred online in 91 cases (6.0%) and face-to-face in 1420 cases (94.0%). The comparison of the two groups is presented in Table 3.

The proportion of female victims was significantly higher in the online-initiated group than in the face-to-face group (89.0% vs. 79.4%; *p* = 0.027). Victim age also differed significantly between the two contact groups (*p* = 0.001; Cramér’s V = 0.107). The proportion of children aged under 9 years was significantly higher in the face-to-face group than in the online-initiated group (18.9% vs. 6.6%), whereas adolescents aged 15–18 years were significantly overrepresented in the online-initiated group (38.5% vs. 23.4%). Overall, 79.2% of the victims in the online-initiated group were aged 12–18 years.

The identity of the perpetrator varied significantly by mode of first contact (*p* < 0.001; Cramér’s V = 0.235). In the online-initiated group, the perpetrator was significantly more likely to be perceived by the child as a boyfriend or friend (81.3% vs. 34.2%). Conversely, the proportions of perpetrators identified as a stranger (25.4% vs. 7.7%), a distant relative (20.6% vs. 9.9%), and a close relative (11.3% vs. 1.1%) were significantly higher in the face-to-face group.

The identity of the reporting person also differed significantly between the groups (*p* = 0.002; Cramér’s V = 0.100). Parents were significantly more often the reporting person in the online-initiated group (47.3% vs. 32.4%), whereas teachers significantly more often reported face-to-face cases (33.5% vs. 15.4%).

Finally, the type of abuse was significantly associated with the mode of first contact (*p* < 0.001; Cramér’s V = 0.148). Cases involving penetrative sexual acts were significantly more prevalent in the online-initiated group (54.9% vs. 26.9%), corresponding to an approximately twofold increase (*RR* = 2.04), whereas non-penetrative cases were more common in the face-to-face group (73.1% vs. 45.1%).

## 4. Discussion

In this forensic series of 1511 judicially reported CSA cases, first contact occurred online in a minority of cases (6.0%). Online-initiated cases nevertheless displayed a distinct profile: the victims were predominantly adolescent girls, the child typically perceived the perpetrator as a friend or boyfriend, parents were more often the reporting source, and penetrative abuse was approximately twice as prevalent as in face-to-face cases. At the same time, the observed base rate underscores that the great majority of CSA in this sample was initiated through in-person contact, most often by individuals within the child’s existing social environment, which is consistent with the broader literature emphasizing that most perpetrators are known to the child [4,5,12]. The interpretation that follows is therefore intended to be proportionate to this base rate: online-initiated contact represents a specific, clinically recognizable pathway rather than the dominant mode of CSA.

The mechanisms outlined in this and the next paragraph derive from previous research rather than from the present dataset and are summarized as an interpretive framework for the relational profile and abuse severity observed in the online-initiated group. Online-facilitated abuse is commonly described as a staged process involving initial friendship formation and trust-building, followed by the gradual collection of personal information that may later be exploited for blackmail, threats, manipulation, or the arrangement of in-person contact. This trajectory often includes sexually explicit communication and requests for sexual content, which may culminate in proposals for offline meetings; once such contact occurs, offenses can escalate to sexual assault, abduction, and related crimes [23]. Importantly, digitally mediated harassment and exploitation do not remain confined to virtual spaces and have been shown to extend into offline environments, including school settings [24].

Population-level indicators highlight the magnitude of exposure risk. In South Korea, the number of adolescent victims of cyber sexual crimes increased approximately thirteenfold between 2018 and 2021 [23]. This interval, however, coincided with the COVID-19 pandemic, during which lockdowns and school closures abruptly shifted children’s education and social interaction online and reported online child sexual exploitation rose worldwide; pandemic-driven digital dependency therefore constitutes a major confounding influence on period-based trend estimates and should temper their interpretation [25]. The present data predate this contextual shift. Separately, in Turkey, a national survey conducted in 2024 reported that 91.3% of children aged 6–15 years used the internet and that 86.2% of those aged 11–15 years owned a smartphone [26]. Taken together, these trends describe a digital environment in which opportunities for grooming behaviors to emerge and to transition into offline sexual victimization are substantial.

Reported prevalence rates of online sexual harassment range from 9.6% to 29.8%, with substantial variation according to age and gender [4,20,27,28]. European studies report that up to 18.5% of adolescents have experienced contact with an online predator, with girls significantly more likely than boys to receive sexual advances [27,28]. In the present study, females constituted the majority of cases in both groups, with a significant excess in the online-initiated group; although internet access is near-universal among children in Turkey [26], girls were approximately eight times more frequent than boys among online-initiated victims (89.0% vs. 11.0%). The male victims in this group were too few to support stratified analyses; because disclosure barriers and stigma are particularly pronounced for boys, victimization of boys initiated online may additionally be underrepresented in forensic samples, and male victims warrant dedicated study. The prior literature is mixed in this respect: some studies report no gender differences in cyber-victimization [29], whereas others find higher victimization among girls [16,30]. Early-stage risk identification frameworks for online grooming and sexual solicitation may help clarify such gendered risk patterns [31].

With respect to age, adolescents have been reported to experience a higher risk of harmful online contact than younger children, although findings vary across studies [32,33], and some research has identified no significant age-related differences in cyber-victimization [30,34]. In the present study, victims aged 15–18 years were significantly more frequent in the online-initiated group (38.5% vs. 23.4%), whereas children under 9 years of age were significantly less frequent, indicating a concentration of online-initiated victimization in adolescence and particularly in later adolescence. This pattern may reflect developmental and behavioral characteristics of adolescence, including greater disclosure of personal and intimate information online, increasing peer-status seeking, and experimentation with self-expression and communication [35,36,37]. Some prior work suggests that vulnerability peaks between the ages of 12 and 15, when electronic communication becomes widespread and socially embedded [16,34,38]; the concentration in older adolescents observed here may partly reflect the forensic nature of the sample and the predominance of perceived romantic relationships, which become more common in later adolescence. Given the retrospective and cross-sectional design, these findings should be interpreted as associative rather than causal.

The most salient contextual correlate of online-initiated abuse in this study was the perpetrator’s position within the child’s close social environment: in 81.3% of online-initiated cases, the child regarded the perpetrator as a friend or boyfriend rather than a stranger. This pattern closely mirrors the model described by Wolak and colleagues, in which internet-initiated sex offenses typically involve adult offenders who openly develop relationships with adolescents and in which victims may perceive the relationship as romantic and the sexual contact as consensual at the time [10]. It must be emphasized that such contact is legally and developmentally non-consensual regardless of the child’s perception, and that the perceived-boyfriend pattern is best understood as a manifestation of grooming dynamics in which deceptive emotional bonds are deliberately cultivated [10,11]. Consistent with this interpretation, prior research indicates that known offenders may use online platforms to groom children, disseminate child sexual abuse material, and arrange in-person meetings [3], and that offenders can readily access vulnerable young people through digital environments that reduce barriers to contact and supervision [39]. Psychosocial vulnerabilities, including lower self-esteem and disinhibition, have been associated with cybergrooming victimization and may increase susceptibility to manipulation [28,40,41]. Offenders, in turn, employ a range of interactional strategies that vary according to individual characteristics, situational dynamics, and abusive intent [12,19].

A further interpretive challenge concerns heterogeneity on the perpetrator side and within the contact process itself. The wide dispersion of perpetrator ages in this sample indicates that online-initiated cases may involve both young, peer-aged offenders and considerably older adults, who are likely to differ in criminal history, offending experience, and networking with other offenders; situations in which one minor contacts another and abuse is subsequently reported raise distinct evidentiary and conceptual difficulties and could not be separated in the present dataset because perpetrator criminal records were unavailable. The relevance of these distinctions is underscored by monitoring surveys in the United States showing that roughly one in four minors regarded sharing self-generated sexual imagery with peers as normal for their age in 2021, that older adolescents were about twice as likely as younger children to hold this view, and that the perceived non-consensual resharing of such imagery by close friends increased year over year [42]. Stratifying such cases by perpetrator age and by the peer versus adult status of the initial contact therefore represents an important direction for subsequent studies.

The Turkish sociocultural context may further shape these relational and disclosure dynamics. Premarital sexual relationships are generally discouraged, prolonged co-residence with parents until marriage or employment is common, and children are culturally perceived as vulnerable and in need of protection. Within this context, covert online relationships may develop outside parental awareness, and their discovery by parents may precipitate reporting; this may partly explain why parents accounted for a significantly larger share of reports in online-initiated cases (47.3% vs. 32.4%), whereas teachers detected a larger share of face-to-face cases.

Notably, the prevalence of penetrative abuse in online-initiated cases was approximately twofold higher than in face-to-face cases (*RR* = 2.04; 54.9% vs. 26.9%). One possible explanation, consistent with RAT, is that online platforms may function as settings in which motivated offenders can identify suitable targets while circumventing effective guardianship [13,15], and deceptive emotional bonds formed online may lower resistance and facilitate arranged in-person meetings in unsupervised settings. However, this is not the only plausible account, and a causal pathway cannot be inferred from these data. The severity difference may also partly reflect differential detection and reporting: face-to-face cases were more often detected by third parties such as teachers and may therefore include a larger share of earlier-stage, non-penetrative acts, whereas online-initiated cases may come to official attention only after the relationship has progressed to physical contact. In addition, because the groups differed in sex, age distribution, and perpetrator identity, part of the unadjusted difference may reflect these compositional characteristics rather than the mode of first contact itself.

To clarify these pathways, future research should delineate adolescent risk profiles within an ecological victimization framework, identify platform- and context-specific features that foster deceptive bonding, and examine the psychosocial mechanisms through which online interactions escalate into offline contact, ideally using prospective or linked-record designs. From a clinical and forensic perspective, the present findings support routinely documenting online contact histories during CSA evaluations and the integration of screening for online-initiated relationships into child protection protocols, particularly for adolescent girls.

### Limitations

Several limitations should be acknowledged. First, and most importantly, this study is based on judicially reported forensic cases. Most CSA is never reported to the authorities, and cases that reach forensic evaluation are unlikely to be representative of all CSA; both the observed proportion of online-initiated cases and the severity comparison may therefore be affected by selection and detection biases, and the findings cannot be generalized to unreported abuse.

Second, the comparison is between two modes of first contact rather than between exposed and non-exposed groups, and no causal inference is possible. The marked imbalance in group sizes (*n* = 91 vs. *n* = 1420) and the predominance of female victims may have reduced statistical power for some subgroup analyses and affected the precision of effect estimates, which are unadjusted for the reasons stated in Section 2.5.

Third, the retrospective design limited the range of assessable variables. It was not possible to determine whether the disclosure of personal information directly facilitated physical contact or whether victims had a history of prior abuse. Several contextual factors, including socioeconomic status, parental supervision, and digital literacy, could not be systematically evaluated, and the low frequency of sex trafficking precluded detailed analysis.

Fourth, the substance use variable was derived from documentation in the victims’ case files; the very high positivity rate observed is a consequence of the operational breadth of the underlying administrative entry described in Section 2.4 and should not be interpreted as an estimate of problematic substance use among the victims.

Finally, family interviews were conducted in only 2.4% of cases; this variable was retained solely as descriptive information on CMC procedures and was not used in any inferential analysis. Clinical and psychiatric data were incomplete, and follow-up information was unavailable, limiting the assessment of mental health outcomes.

## 5. Conclusions

In this large forensic sample of judicially reported CSA cases, online-initiated first contact, although present in a minority of cases, was associated with a distinct case profile: victims were predominantly adolescent girls, perpetrators were most often perceived as friends or boyfriends, and the proportion of penetrative abuse was about double that of face-to-face cases. These findings are associative and should not be interpreted as evidence that online contact causes more severe abuse; rather, they identify online-initiated contact as a clinically and forensically relevant contextual marker. Systematic documentation of online interaction histories during CSA evaluations may enhance risk recognition, support forensic assessment, and inform prevention strategies that integrate digital-context awareness into child protection frameworks.

## Figures and Tables

**Table 1:** Characteristics of sexual abuse in the overall sample (*N* = 1511).

Variable	Category	*n*	%
Identity of the perpetrator	Boyfriend/friend	560	37.1
	Stranger	367	24.3
	Other relative (uncle, cousin, grandfather)	302	20.0
	Close relative (father, brother, stepfather, stepbrother)	162	10.7
	Babysitter	120	7.9
More than one suspect	No	1330	88.0
	Yes	181	12.0
Place of incident	Home of the victim	199	13.2
	Home of the perpetrator/indoor environment	491	32.5
	School or social areas	821	54.3
Type of abuse	Without penetration	1079	71.4
	With penetration	432	28.6
Pregnancy	No	1374	90.9
	Yes	137	9.1
Sex trafficking	No	1498	99.1
	Yes	13	0.9
Substance use (victim, as documented in case files) *	No	85	5.6
	Yes	1426	94.4
Disability	Absent	1455	96.3
	Present	56	3.7

* Reflects documentation in institutional forensic records rather than clinically verified problematic substance use.

**Table 2:** Summary of the procedures applied at the Child Monitoring Center (*N* = 1511).

Variable	Category	*n*	%
Temporary accommodation in the center	No	1481	98.0
	Yes	30	2.0
Forensic interview	Applied	1374	90.9
	Not applied	137	9.1
Family interview	Applied	36	2.4
	Not applied	1475	97.6
Procedures applied in the center	Forensic examination only	140	9.3
	Forensic examination + mental health evaluation	1024	67.8
	Pregnancy test	331	21.9
	Bone age determination	16	1.1
Procedures applied with support agencies/institutions	The child was taken into institutional care	215	14.2
	Institutional care was ongoing	113	7.5
	Social services evaluation was requested	252	16.7
	No additional institutional procedure	931	61.6

**Table 3:** Comparison of parameters between the online-initiated contact group and the face-to-face contact group.

Variable	Category	Online-Initiated *n*	%	Face-to-Face *n*	%	Total *n*	%	*p* Value
Gender of the victim	Female	81	89.0 ^a^	1128	79.4 ^b^	1209	80.0	0.027
	Male	10	11.0 ^b^	292	20.6 ^a^	302	20.0	
Age of the victim	<9 years	6	6.6 ^b^	268	18.9 ^a^	274	18.1	0.001
	9–12 years	13	14.3 ^a^	297	20.9 ^a^	310	20.5	
	12–15 years	37	40.7 ^a^	523	36.8 ^a^	560	37.1	
	15–18 years	35	38.5 ^a^	332	23.4 ^b^	367	24.3	
Identity of the perpetrator	Boyfriend/friend	74	81.3 ^a^	486	34.2 ^b^	560	37.1	<0.001
	Stranger	7	7.7 ^b^	360	25.4 ^a^	367	24.3	
	Other relative (uncle, cousin, grandfather)	9	9.9 ^b^	293	20.6 ^a^	302	20.0	
	Close relative (father, brother, stepfather, stepbrother)	1	1.1 ^b^	161	11.3 ^a^	162	10.7	
	Babysitter	0	0.0 ^b^	120	8.5 ^a^	120	7.9	
Reporting person	Parent	43	47.3 ^a^	460	32.4 ^b^	503	33.3	0.002
	Teacher	14	15.4 ^b^	476	33.5 ^a^	490	32.4	
	Victim	13	14.3 ^a^	155	10.9 ^a^	168	11.1	
	Other (healthcare personnel, police/gendarmerie, relatives, neighbors)	21	23.1 ^a^	329	23.2 ^a^	350	23.2	
Type of abuse	Without penetration	41	45.1 ^b^	1038	73.1 ^a^	1079	71.4	<0.001
	With penetration	50	54.9 ^a^	382	26.9 ^b^	432	28.6	

Note: Different superscript letters within a row indicate a statistically significant difference between column proportions according to Bonferroni-adjusted post hoc tests; the proportion labeled “a” is greater than the proportion labeled “b”.

## Data Availability

The data presented in this study are available on reasonable request from the corresponding author. The data are not publicly available due to the sensitive nature of forensic records concerning minors and applicable privacy and ethical restrictions.
